# Quantification of Interfibrillar Shear Stress in Aligned Soft Collagenous Tissues via Notch Tension Testing

**DOI:** 10.1038/srep14649

**Published:** 2015-10-15

**Authors:** Spencer E. Szczesny, Jeffrey L. Caplan, Pal Pedersen, Dawn M. Elliott

**Affiliations:** 1 Department of Bioengineering. University of Pennsylvania, 240 Skirkanich Hall, 210 South 33rd St, Philadelphia, PA 19104; 2 Delaware Biotechnology Institute, 15 Innovation Way, Suite 117, Newark, DE 19716; 3 Carl Zeiss Microscopy, One Zeiss Drive, Thornwood, NY 10594; 4 Department of Biomedical Engineering, University of Delaware, 161 Colburn Lab, 150 Academy Street, Newark, DE 19716

## Abstract

The mechanical function of soft collagenous tissues is largely determined by their hierarchical organization of collagen molecules. While collagen fibrils are believed to be discontinuous and transfer load through shearing of the interfibrillar matrix, interfibrillar shear stresses have never been quantified. Scaling traditional shear testing procedures down to the fibrillar length scale is impractical and would introduce substantial artifacts. Here, through the use of a novel microscopic variation of notch tension testing, we explicitly demonstrate the existence of interfibrillar shear stresses within tendon fascicles and provide the first measurement of their magnitude. Axial stress gradients along the sample length generated by notch tension testing were measured and used to calculate a value of 32 kPa for the interfibrillar shear stress. This estimate is comparable to the interfibrillar shear stress predicted by previous multiscale modeling of tendon fascicles, which supports the hypothesis that fibrils are discontinuous and transmit load through interfibrillar shear. This information regarding the structure-function relationships of tendon and other soft collagenous tissues is necessary to identify potential causes for tissue impairment with degeneration and provide the foundation for developing regenerative repair strategies or engineering biomaterials for tissue replacement.

Soft collagenous tissues (e.g., tendon, ligament, annulus fibrosus, meniscus, arteries, cardiac valves) are primarily composed of collagen fibrils, which consist of a semi-crystalline organization of type I collagen molecules connected through naturally occurring inter-molecular crosslinks^1^,^2^,^3^. While the specific organization of suprafibrillar structures varies with tissue type and has important implications on tissue mechanics^4^, the fundamental fibrillar deformation mechanisms and interfibrillar interactions that underlie the mechanical properties of these tissues are unknown. Previous multiscale investigations suggest that the collagen fibrils in these tissues are discontinuous and that load is transferred between fibrils through their relative sliding and shearing of the interfibrillar matrix^5^,^6^,^7^,^8^. Furthermore, plastic deformation of the interfibrillar matrix, rather than failure of the fibrils themselves, has been suggested to be the failure mechanism responsible for tissue post-yield behavior^9^,^10^. However, no experimental techniques are available to confirm the existence of interfibrillar shear stress within intact tissues or to directly measure their magnitude. Such information is necessary to conclusively test these structure-function hypotheses and identify changes in the hierarchical deformation mechanisms that impair tissue function and promote failure with disease or degeneration.

Notch tension testing, an approach typically used to evaluate crack propagation and fracture toughness^11^, provides an opportunity to overcome the limitations of existing technologies and measure interfibrillar shear stress. While traditional shear testing procedures have been applied to macroscopic sections of human ligaments^12^, scaling these experiments down to the fibrillar length scale is impractical and would introduce substantial artifacts from gripping the tissue in close proximity to the region of interest. Alternatively, pullout testing of individual fibrils has been successfully conducted on antler bone in a combined AFM-SEM experimental setup^13^; however, these tests require fracturing the tissue and cannot be applied to non-mineralized fibrous tissues due to rapid dehydration under the vacuum conditions. In contrast, notch tension testing requires no specialized experimental setup and can be performed using the same conditions employed for standard uniaxial tension^5^. By combining notch tension testing and confocal microscopy, we demonstrated the existence of interfibrillar shear stresses within intact tendon fascicles and calculated their magnitude in a fully hydrated environment. Interestingly, the calculated values are comparable to the interfibrillar shear stress predicted by shear lag modeling of tendon fascicles^5^, which suggests that these models accurately describe tendon fascicle multiscale mechanics. Similar techniques can be applied to other aligned soft collagenous tissues to identify differences in interfibrillar shear stress with tissue structure or degeneration. The discovery and quantification of these structure-function relationships is necessary to identify potential causes for tissue impairment with degeneration and provides the foundation for developing regenerative repair strategies or for engineering biomaterials for tissue replacement.

## Results

### Testing of Gelatin Gel

**Figure 1:**
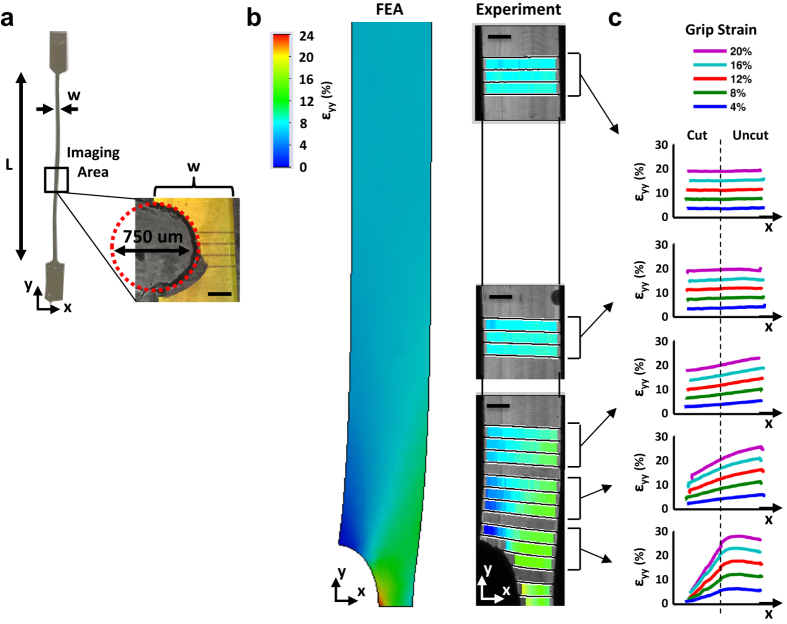
Demonstration of notch tension technique using a linear elastic isotropic gelatin gel. (**a**) Uniaxial tension was applied to a 20% (w/v) gel formed with similar dimensions as a tendon fascicle (h: 540 μm, w: 680 μm, L: 33.0 mm) and with a semi-circular notch. (**b**) At a grip-to-grip strain of 8%, the axial strain field (ε_yy_) predicted by finite element analysis closely matched the experimentally measured strains. (**c**) Close to the notch, the axial strains are concentrated on the uncut (right) side of the gel, while the strains are uniform far from the notch. These gradients in the axial strain (and hence stress) across the gel width and length are classic features of notch tension testing and are produced by shear stresses that transmit load from the uncut to the cut side of the sample. Scale bars, 200 μm.

To demonstrate the accuracy of our notch tension technique and provide a proof-of-concept for the experimental analysis, we tested a 20% (w/v) gelatin gel containing a semi-circular notch (Fig. 1a) on a custom uniaxial testing device mounted on a confocal microscope^5^. Lines photobleached onto the gel surface were used to measure the axial strain (ε_yy_) under the microscope at multiple locations along the sample length. At a grip-to-grip strain of 8%, the measured axial strain field matches that predicted by a separate finite element analysis (Fig. 1b), which demonstrates that the technique of tracking photobleached lines can accurately measure the axial strain distributions at the microscopic level. For all grip-to-grip strains, a gradient in the axial strain across the gel width is observed at the locations closest to the notch, with strains on the uncut (right) side of the gel greater than the applied grip-to-grip strain whereas the strains on the cut (left) side are less than the applied value (Fig. 1c). Furthermore, a gradient in the axial strain also exists along the gel length. This is demonstrated by the fact that the strains on the uncut side of the gel decrease with distance away from the notch while the strains on the cut side increase. Ultimately, at the locations far from the notch, the axial strains have equilibrated across the gel width producing a uniform strain distribution equal to the applied deformation.

These changes in the axial strain (and hence stress) distributions in the linear elastic isotropic gel are a classic feature of notch tension testing and are produced by shear stresses that transmit load from the uncut to the cut side of the sample. This relationship between the shear stress (*σ*_*xy*_) and axial stress gradient along the sample length (*dσ*_*yy*_* /dy*) is described by the axial force equilibrium equation (i.e., *dσ*_*yy*_* /dy* + *dσ*_*xy*_* /dx* = *0*). In the absence of shear stress (*dσ*_*xy*_* /dx* = *0*), the axial stress (and strain) distribution will not change with distance from the notch (i.e., *dσ*_*yy*_*/dy* = *dε*_*yy*_* /dy* = 0) (Supplementary Fig. 1). However, as seen in Fig. 1c, there is indeed an axial strain gradient along the length of the gelatin gel (*dε*_*yy*_* /dy* ≠ 0), demonstrating that non-zero shear stresses exist within the material. Therefore, by measuring the change in the axial stress with distance from the notch (*dσ*_*yy*_* /dy*), we can determine whether shear stresses are generated within a tissue or biomaterial. Furthermore, as we will show for tendon fascicles, the magnitude of the axial stress gradient along the sample length produced by notch tension testing can be used to estimate the value of the interfibrillar shear stress acting within an aligned soft collagenous tissue.

### Testing of Tendon Fascicles

**Figure 2:**
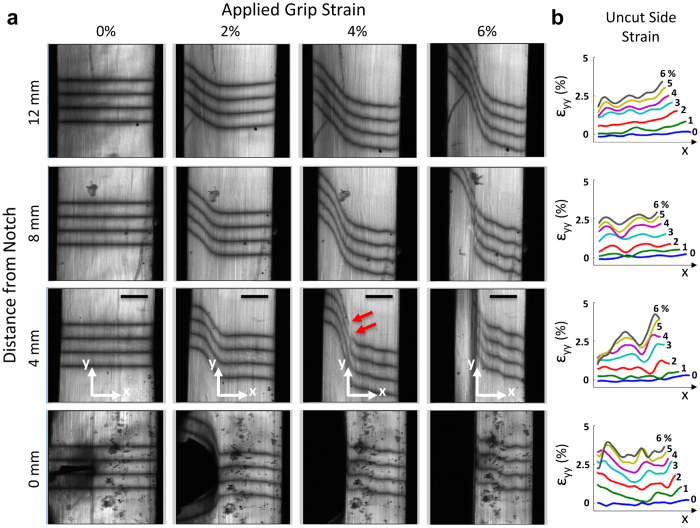
Notch tension testing of tendon fascicles. (**a**) With increasing grip-to-grip strain, the notch widened and the photobleached lines became steeply angled, representing shear strains developing within the tissue. Consistently at 4% grip-to-grip strain, discontinuities in the photobleached lines first appeared at the location 4 mm from notch (red arrows). These discontinuities propagated parallel to the fascicle axis demarcating the interface between the cut and uncut portions of the tissue. (**b**) At all non-zero distances from the notch, the axial strains (ε_yy_) on the uncut side of the tissue exhibit gradients similar to those seen in the gel (Fig. 1c). Scale bars, 200 μm.

**Figure 3:**
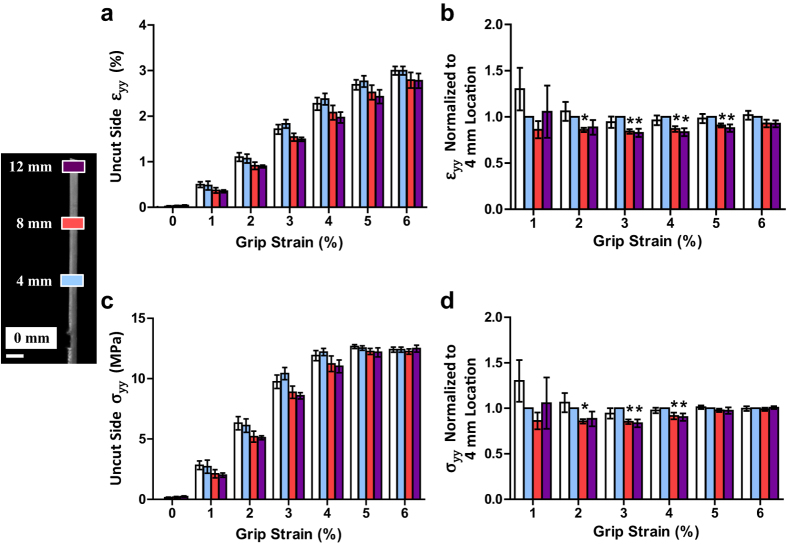
Axial strain and stress gradients produced in tendon fascicles. (**a**,**b**) The axial strains (ε_yy_) averaged over the uncut side of the tissue decreased with distance away from the notch. This axial strain gradient along the sample length is similar to that observed on the uncut side of the gelatin gel and demonstrates the existence of shear stress within the tissue. (**c**,**d**) Conversion of these strains to stress values shows that the axial stress (σ_yy_) on the uncut side of the tissue decreased with distance away from the notch until 4% grip-to-grip strain. This drop in axial stress corresponded to an interfibrillar shear stress of 32 ± 33 kPa (mean ± s.d.). *p ≤ 0.05. Error bars, s.e.m. Scale bar, 1 mm.

In order to specifically investigate the existence of interfibrillar shear stresses, we applied our microscale notch tension technique to rat tail tendon fascicles. Because this tissue is composed of highly aligned collagen fibrils^14^, the shear stresses that are produced will primarily be due to relative sliding of the fibrils and shearing of the interfibrillar matrix^5^,^6^ as opposed to fibrillar rotation and realignment that would occur in less organized materials (e.g. collagen gels). Microscopic images taken at the notch location and along the fascicle length show that strain applied to the tissue widens the notch and generates steep angles in the photobleached lines, which represents large shear strains within the fascicle (Fig. 2a). These shear strains increase until an applied 4% grip-to-grip strain, at which point a discontinuity in the photobleached lines appears at the location closest to the notch. Further applied strain causes the discontinuity to propagate along the tissue length away from the notch and parallel to the fascicle longitudinal axis. The formation and propagation of these discontinuities suggests that the notch tip is being deflected by 90°, thereby preventing any lateral progression of the notch across the sample width. Furthermore, the near perfect alignment of the propagation along the fascicle axis (Supplementary Fig. 2) demarcates the interface between the cut and uncut portions of the fascicle. Calculation of the axial strain (ε_yy_) on the uncut side (i.e., to the right of the discontinuity interface) demonstrates the existence of strain gradients across the fascicle width (Fig. 2b) that are similar but smaller in magnitude to those seen in the gelatin gel. Averaging the axial strains over the uncut side of the fascicle demonstrated that there was a consistent decrease in the axial strains with distance away from the notch (Fig. 3a,b). Again, this axial strain gradient is similar to that observed on the uncut side of the gelatin gel, and as explained above, demonstrates the existence of shear stress within the tissue.

In order to estimate the magnitude of the shear stresses, we converting the averaged axial strains to stresses using empirical non-linear stress-strain data collected from previous testing of intact tendon fascicles (Supplementary Fig. 3)^15^ and quantified the axial stress gradient along the fascicle length (Fig. 3c,d). This assumed that the strains of the collagen fibrils are the dominant source of axial stress within the fascicle and that the contributions of the lateral strain (ε_xx_) or shear strain (ε_xy_) of the interfibrillar matrix can be neglected, which is common for tendon and other soft collagenous tissues^16^,^17^. Interestingly, the axial stress diminished with distance away from the notch until 4% grip-to-grip strain, after which no axial stress gradient was observed. This is consistent with the first appearance of discontinuities in the photobleached lines (Fig. 2a), which suggests that the discontinuities represent a failure of the cut/uncut tissue interface, thereby eliminating the shear stress at this interface after failure. Furthermore, no strain or stress gradients along the sample length were measured during testing of intact samples (Supplementary Fig. 4), demonstrating that this phenomenon is due to the shear stress within the tissue and not gripping artifacts. After converting the equilibrium equation to an axial force balance (see Supplementary Methods and Supplementary Fig. 5), we used the measured axial stress gradients to calculate an average shear stress of 32 ± 33 kPa (mean ± s.d.) prior to failure at the cut/uncut tissue interface.

### Tracking of Fibril Trajectories

There is evidence to suggest that the shear stress value calculated above is representative of the interfibrillar shear stress produced by relative sliding of fibrils and shearing of the interfibrillar matrix. Prior to the discontinuity initiation and propagation, the photobleached lines were continuous down to the limiting resolution of the microscope (Supplementary Fig. 6). This suggests that the observed shear deformations and measured shear stress occur at a length scale below the imaging resolution (i.e., between the individual fibrils). Furthermore, the shear stress value of 32 kPa is within the bounds of the interfibrillar shear strength in bone^13^ and comparable to the interfibrillar shear stress predicted by multiscale mechanical models of tendon^5^. Nevertheless, a primary assumption supporting this interpretation is that the collagen fibrils are highly aligned with the fascicle axis and that the observed axial stress gradient along the sample length is not due to angled fibrils crossing the cut/uncut tissue interface.

**Figure 4:**
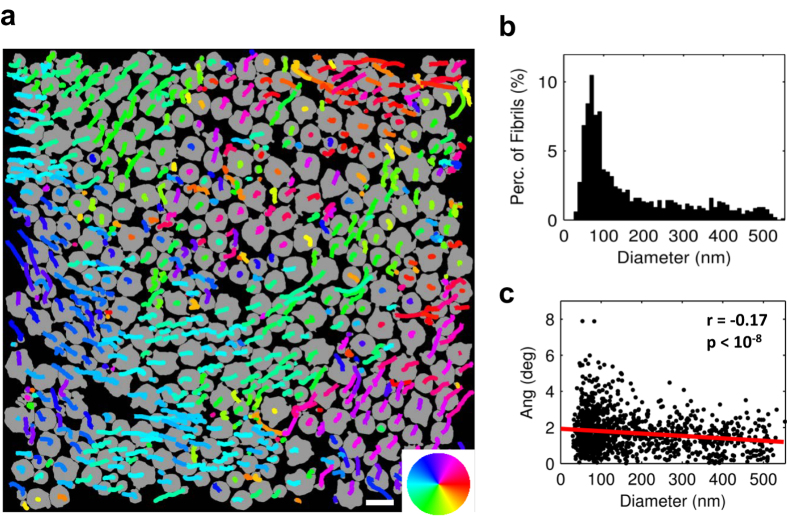
Trajectories of individual collagen fibrils. (**a**) Fibrils (shown in gray) from first image with overlay of lateral trajectories along 8.7 μm of fascicle length. Color wheel indicates direction of lateral trajectory. (**b**) Histogram of fibril diameters. (**c**) While there was a weak correlation between angle and fibril diameter (r = −0.17, p < 10^−8^), on average the fibrils were oriented 1.7 ± 1.0 deg (mean ± s.d.) with respect to the fascicle axis, demonstrating that the fibrils are highly aligned within the fascicles and suggesting that few fibrils cross the cut/uncut tissue interface. Scale bar, 500 nm.

To verify this assumption and confirm that the axial stress gradient was indeed due to interfibrillar shear stress, we tracked the course of 1,213 individual fibrils by serially imaging cross-sections of a tendon fascicle over a length of 8.7 μm using serial block-face scanning electron microscopy^18^. This allowed us to determine the lateral trajectories of the fibrils along the fascicle length and calculate their angles with respect to the fascicle axis (Fig. 4). We found that the fibrils were closely aligned with the fascicle axis, with an average angle of 1.7 ± 1.0 deg (mean ± s.d.) and a weak correlation between angle and fibril diameter (r = −0.17, p < 10^−8^). These data are consistent with previous X-ray diffraction data^19^ and the observation that the discontinuities in the photobleached lines propagated parallel to the fascicle axis (Fig. 2a and Supplementary Fig 2). This suggests that few fibrils cross the cut interface and that the axial stress gradient is indeed due to interfibrillar shear stress.

## Discussion

In this study, we used microscale notch tension testing to make the first explicit demonstration and measurement of interfibrillar shear stresses within a soft collagenous tissue. We found that interfibrillar shear stresses in rat tail tendon fascicles reach approximately 32 kPa prior to failure at the cut/uncut tissue interface. This value is comparable to that predicted by shear lag modeling of the same tissue^5^, which further supports the hypothesis that fibrils are discontinuous and transmit load through interfibrillar shear. Additionally, our notch tension technique demonstrated that tendon fascicles are insensitive to the introduction of a local tissue defect. We observed in all samples that the notch tip deflected by 90° and propagated longitudinally along the fascicle length. This is a classic toughening mechanism for fiber-reinforced composite materials^20^, where fracture along the fibril-matrix interface isolates damage accumulation to within the interfibrillar matrix, thereby preventing lateral growth of the crack tip across the fascicle width and rupture of the load-bearing fibrils. Furthermore, this behavior is consistent with previous studies suggesting that plastic deformation of the interfibrillar matrix, rather than failure of the fibrils themselves, is the mechanism underlying yielding of intact tendon fascicles under uniaxial tension^9^,^10^. Similar crack deflections and lack of crack tip lateral growth is also observed at the whole tendon level^21^,^22^, which suggests that the inter-*fascicular* matrix protects against tendon rupture due to failure of individual fascicles^23^. Such hierarchical repetition of toughening mechanisms across multiple length scales is also observed in bone and has been shown to exponentially enhance the flaw tolerance of the entire tissue^24^. Identification of these failure mechanisms and hierarchical structure-function relationships is essential for discovering the underlying causes of tissue impairment with disease, for guiding regenerative repair strategies to restore tissue function, and for engineering biomaterials that can effectively replace native tissues.

A limitation to the techniques described in this study is that the calculation of the interfibrillar shear stress is limited to soft collagenous tissues with highly aligned fibrils. While notch tension testing will still produce shear stresses in less organized tissues, these shear stresses will result from rotation and realignment of the collagen fibrils as well as from interfibrillar shear. Additionally, it was necessary to assume that only the fibril strains contribute to the axial stress within the fascicle in order to convert the measured axial strains into stress values. While this is acceptable for tendon^16^,^25^, it is not valid for fibrocartilaginous tissues with a stiffer interfibrillar matrix (e.g., cartilage, meniscus). In these cases, constitutive modeling of the tissue mechanics would be necessary to analyze the strain fields and interpret a value for the interfibrillar shear stress.

We chose not to use a constitutive model and instead used a simplified analysis involving a finite difference approximation based on the equations of equilibrium (see Supplementary Methods) for a couple reasons. First, an objective of this study was to validate the predicted interfibrillar shear stress values from previous multiscale modeling of tendon^5^. Therefore, we did not prescribe a particular constitutive behavior for the tissue in order to ensure that the value obtained in the current work is independent of the model choice. Second, due to the high tensile modulus of rat tail tendon fascicles, the axial stress gradients produced by the notch correspond to small strain gradients that are of similar magnitude to the inherent noise in the microscale strain measurements. This is evident in the oscillatory plots of the axial strains in the tendon fascicles (Fig. 2b), which contrasts the straight (non-oscillatory) plots for the softer gelatin gel (Fig. 1c). Therefore, we chose an analysis technique that provided an average value for the interfibrillar shear stress at the cut/uncut interface rather than use a more sophisticated analysis (i.e., inverse finite element methods) that would be sensitive to measurement noise. Nevertheless, the current technique produced an interfibrillar shear stress value that is comparable to previous work^5^,^13^, which provides valuable insight into tendon structure-function relationships and supports the hypothesis that fibrils in soft collagenous tissues are discontinuous and transmit load through interfibrillar shear.

## Methods

See Supplementary Methods for full details.

### Testing of gelatin gel

#### Sample preparation

A 20% (w/v) gelatin gel was crosslinked with 0.05% glutaraldehyde in phosphate buffered saline (PBS) in order to prevent swelling and stabilize the mechanical properties^26^. The gel was then fluorescently stained with 10 μg/ml of 5-(4,6-dichlorotriazinyl)aminofluorescein (Life Technologies), washed in PBS, and cut with a 0.75 mm biopsy punch to produce a semi-circular notch. The gel was then loaded into the PBS bath of a custom uniaxial testing device mounted on an inverted confocal microscope^5^ (LSM 5 LIVE; 10× C-Apochromat water immersion lens, Zeiss).

#### Mechanical Testing

Prior to testing, a 1 mN preload was applied to define the reference length (33.0 mm). Sets of four lines were photobleached onto the gel surface at 0, 0.4, 0.8, 1.2, 2.0, and 4.0 mm from the notch midline. Grip-to-grip strains were applied to the gel in 4% strain increments at 0.05%/s and held for 5 min before imaging the photobleached lines at each location. This was repeated to a total grip-to-grip strain of 20%. The applied load was continuously recorded with a 10 N load cell (Model 31, Honeywell).

*Data analysis*. A custom Matlab algorithm was used to find the pixel locations of the photobleached lines in each image at all locations. Axial strains (ε_yy_) were calculated as the change in the distance between line pairs compared to their positions at 0% applied strain. The Young’s modulus of the gel (*E* = 585 kPa) was determined by performing a least-squares fit of the line *σ* = *Eε*, where σ is the applied stress and ε is the average axial strain at the 4 mm location measured for each grip-to-grip strain increment (R^2^ = 0.993). Note that 4 mm was suitably far-field from the notch so that the axial strains at this location were uniform (Fig. 1c).

#### Finite element analysis

The finite element model was constructed within SolidWorks (Dassault Systemes) with the same geometry as the imaging area of the physical gel sample and composed of triangular two-dimensional shell elements. A fixed constraint was applied at a distance 5 mm from the notch and a 0.4 mm displacement was applied to the notch midline, simulating an applied tensile strain of 8%. The material was defined as isotropic, linear elastic, and incompressible with a Young’s modulus of 585 kPa. The resulting axial strain field was compared graphically to the experimental measurements taken at a grip-to-grip strain of 8%.

### Testing of tendon fascicles

#### Sample preparation

Eight fascicles were harvested from five tails of 7-month-old Sprague-Dawley rats sacrificed in accordance with IACUC approval and relevant guidelines. Each fascicle was cut to a length of 45 mm and fluorescently stained with 10 μg/ml of 5-(4,6-dichlorotriazinyl)aminofluorescein. Previous testing of intact samples demonstrated that staining at this concentration has a minimal effect on tendon fascicle mechanics^15^. The samples were washed in PBS and placed into the same uniaxial device used to test the gel construct. The sample cross-sectional area was measured using the confocal microscope (see Supplementary Methods).

#### Mechanical Testing

A 1 mN preload (~5 kPa) was applied to the tissue to define the reference length (31.9 ± 0.1 mm). The sample was preconditioned and then partially transected with a scalpel blade 8.7 ± 0.3 mm from either the left or right grip producing a sharp notch extending 48 ± 13% across the sample width. The notch was placed near each grip for an equal number of samples to ensure that the measured strain gradients were due to the presence of the notch and not gripping artifacts. Sets of four lines were photobleached onto the tissue surface at 0, 4, 8, and 12 mm from the notch. Grip-to-grip strains were then applied in 1% strain increments at 0.05%/s and held for 5 min before capturing image stacks (0.82 × 0.82 × 3.7 μm/pixel) of the photobleached lines at each location. This was repeated to a total grip-to-grip strain of 8%. Image stacks (80–100 images spanning 300–350 μm) were obtained since the curved surface of the fascicles meant that no single focal plane contained an image spanning the full tissue width. After testing, the microscale image stacks were flattened to a single image of the full tissue width using a custom Matlab algorithm (see Supplementary Methods and Supplementary Fig. 7). Using this flattened image, the pixel locations of the photobleached lines and the axial strains (ε_yy_) were calculated as described for the gel construct.

#### Calculation of axial stress gradient and interfibrillar shear stress

Based on the axial force equilibrium equation *∂σ*_*yy*_* /∂y* + *∂σ*_*xy*_* /∂x* = *0*, an estimate for the interfibrillar shear stress (*σ*_*xy*_) was calculated from the axial stress gradient (*∂σ*_*yy*_* /∂y*) on the uncut side of the fascicle. The boundary of the uncut side was determined as the x-position of the initial discontinuity within the photobleached lines at each imaging location (Fig. 2a). These positions were also used to determine the propagation angle of the discontinuities along the sample length with respect to the fascicle axis (Supplementary Fig. 2). Assuming that the uncut side was a constant fraction of the fascicle width throughout testing, the position of the uncut boundary at earlier points during testing was determined at each imaging location using the width fraction computed from the initial appearance of discontinuity. To reduce the effect of noise on the shear stress calculation, the axial strains were averaged across the width of the uncut side for every applied grip-to-grip strain value and at every imaging location. Assuming that only the axial strains of the fibrils contribute to the axial stress within the fascicle and neglecting any contribution from transverse or shear strains of the matrix^16^,^25^, the average axial strain on the uncut side was converted to stress using an empirical relationship between the microscale fibril strains and applied stress from previous testing of intact rat tail tendon fascicles using the same testing protocols^15^ (Supplementary Fig. 3). To evaluate the stress gradient within each sample, the stress values were normalized to the location 4 mm away from the notch. Normalized values statistically different from one were determined by one sample Student’s t-tests with significance set at p ≤ 0.05.

The interfibrillar shear stress was calculated as the average shear stress acting at the interface between the cut and uncut sides of the tissue. Shear stress values (*σ*_*xy*_) were computed using the axial force balance *σ*_*xy*_* A*_*x*_ = Δ*σ*_*yy*_* A*_*y*_, where Δ*σ*_*yy*_ is the decrease in axial stress between the 4 mm and 8 mm locations or the 8 mm and 12 mm locations at each grip-to-grip strain, *A*_*x*_ is the interfacial area between the cut and uncut sides, and *A*_*y*_ is the average uncut cross-sectional area (Supplementary Fig. 5a). Note that this force balance can be derived directly from the axial force equilibrium equation (see Supplementary Methods). Shear stress values were calculated at each grip-to-grip strain up to the initiation of failure at the cut/uncut tissue interface (i.e., 4% strain) and were averaged to produce a single value for the interfibrillar shear stress acting within each sample.

### Tracking of fibril trajectories

One fascicle was harvested from the tail of a 7-month-old Sprague-Dawley rat and cut into segments approximately 10 mm long. According to a protocol specifically developed for tracking collagen fibrils in tendon using serial block-face scanning electron microscopy (SBF-SEM)^18^, the segments were fixed with glutaraldehyde, stained with osmium tetroxide, tannic acid, and uranyl acetate, dehydrated, and embedded in EMbed 812 Hard resin (Electron Microscopy Sciences). The resin block was positioned with the fascicle axis oriented vertically and then faced to expose the tissue cross-section. At a single location, 88 serial cross-sectional images (2000 × 2000 pixels, 5 nm/pixel) were taken every 100 nm along the fascicle length with a Merlin VP Compact scanning electron microscope (Zeiss) fitted with a 3View2XP *in situ* ultramicrotome (Gatan). The raw images were processed using Fiji^27^ and the fibrils were tracked with a custom Matlab algorithm (see Supplementary Methods).

## Additional Information

**How to cite this article**: Szczesny, S. E. *et al*. Quantification of Interfibrillar Shear Stress in Aligned Soft Collagenous Tissues via Notch Tension Testing. *Sci. Rep*. **5**, 14649; doi: 10.1038/srep14649 (2015).

## Supplementary Material

Supplementary Information
