# Supplementary material for: Quantification of Interfibrillar Shear Stress in Aligned Soft Collagenous Tissues via Notch Tension Testing
Source: Sci Rep. 2015 Oct 15;5:14649. doi: 10.1038/srep14649 (PMC4606738; doi:10.1038/srep14649)
Supplement: Supplementary Information [file srep14649-s1.pdf]

Quantification of Interfibrillar Shear Stress in Aligned Soft Collagenous Tissues via Notch Tension Testing

Spencer E. Szczesny[a], Jeffrey L. Caplan[b], Pal Pedersen[c], Dawn M. Elliott[d*]

[a]Department of Bioengineering
University of Pennsylvania
240 Skirkanich Hall
210 South 33rd St
Philadelphia, PA 19104
esy@seas.upenn.edu

[b]Delaware Biotechnology Institute
15 Innovation Way, Suite 117
Newark, DE 19716
jcaplan@udel.edu

[c]Carl Zeiss Microscopy
One Zeiss Drive
Thornwood, NY 10594
pal.pedersen@zeiss.com

[d]Department of Biomedical Engineering
University of Delaware
161 Colburn Lab
150 Academy Street
Newark, DE 19716
delliott@udel.edu

*Corresponding Author:
Dawn M. Elliott, Ph.D.
161 Colburn Lab
150 Academy Street
Newark, DE 19716
Phone: 302-831-1295
delliott@udel.edu

Supplementary Figures

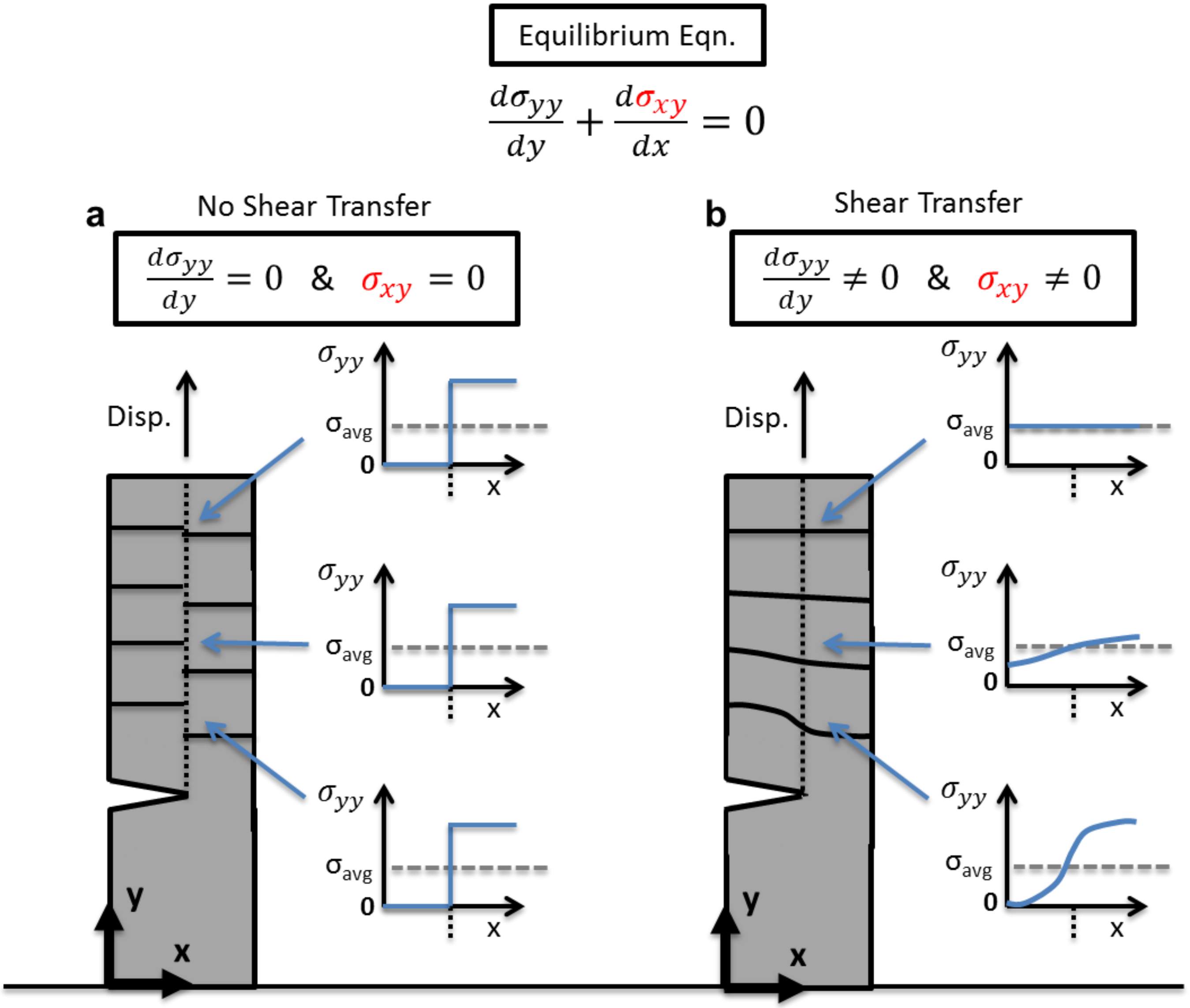

Supplementary Figure 1. Conceptualization of shear stress calculation using notch tension testing. (a) In a material that is incapable of generating shear stresses, the distribution of axial stress along the sample width does not change with distance from the notch. (b) If the material supports shear load transfer, based on the axial force equilibrium equation, measurement of the axial stress gradient along the sample length can be used to calculate the magnitude of the shear stress acting within the tissue or biomaterial.

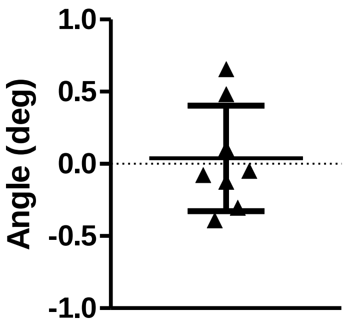

Supplementary Figure 2. Angle of crack propagation. The discontinuities in the photobleached lines propagated at angles near zero with respect to the fascicle axis. Error bars, s.d.

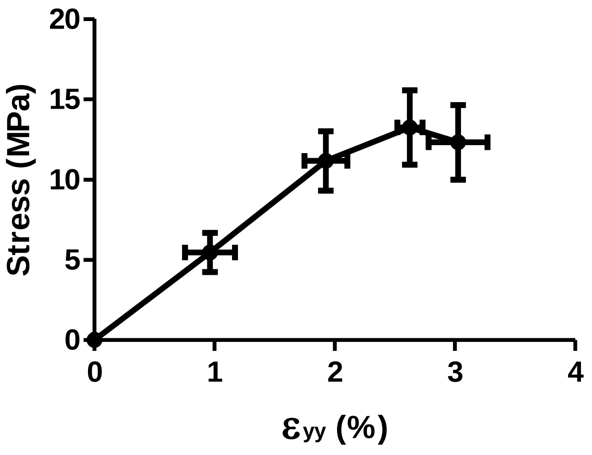

Supplementary Figure 3. Stress-strain curve of intact tendon fascicles. Data from prior testing of intact tendon fascicles[15] was used to convert the microscopic axial strains measured during notch tension testing into stress values. Error bar, s.d.

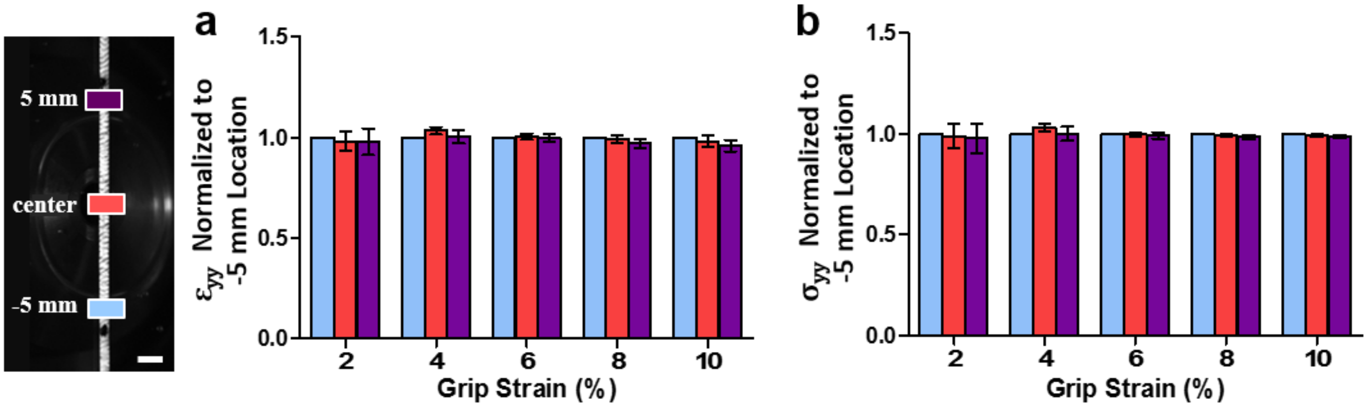

Supplementary Figure 4. Lack of stress gradient during intact testing. (a,b) The axial strain (ε_{yy}) and axial stress (σ_{yy}) within intact tendon fascicles (normalized to the location -5 mm from the sample midpoint) is uniform over the sample length. This is in contrast to the stress gradients observed during notch tension testing, demonstrating that those gradients are due to the notch itself and not gripping artifacts. Error bars, s.e.m. Scale bar, 1 mm.

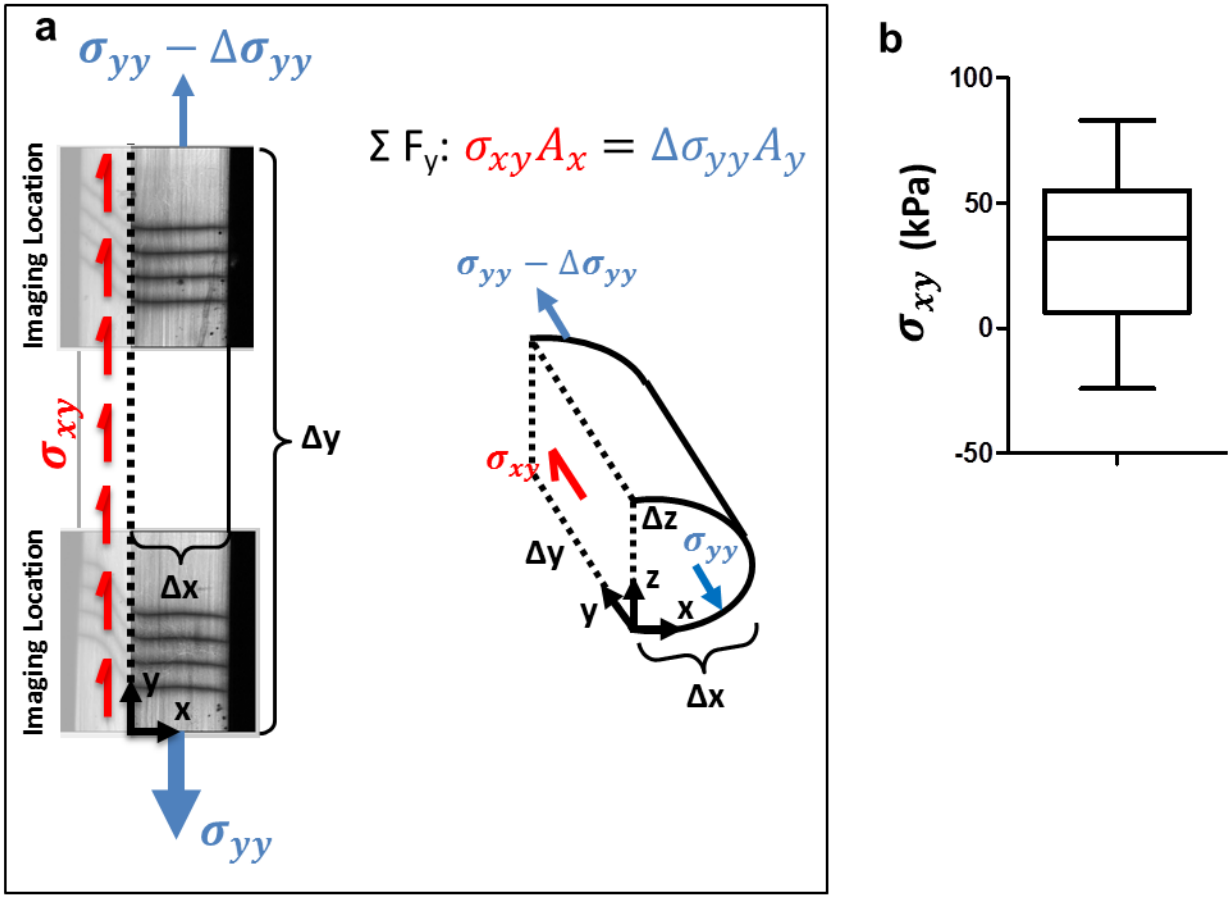

Supplementary Figure 5. Calculation of shear stress. (a) Schematic of the axial force balance (ΣF_y) used to calculate the equivalent shear stress (σ_{xy}) corresponding to the measured axial stress (σ_{yy}) gradients. The shear stress acting at the cut/uncut tissue interface ($A_x = \Delta y \Delta z$) is balanced by the decrease in the axial stress ($\Delta\sigma_{yy}$) between the imaging locations acting over the intact cross-sectional area ($A_y \sim \Delta x \Delta z$). (b) Boxplot of the average calculated shear stress prior to failure at the cut/uncut tissue interface. Note that σ_{xy} in the figure represents $\sigma_{xy}(0, y_i)$ in the derivation of the axial force balance (see Supplementary Methods). Figure drawn by SES.

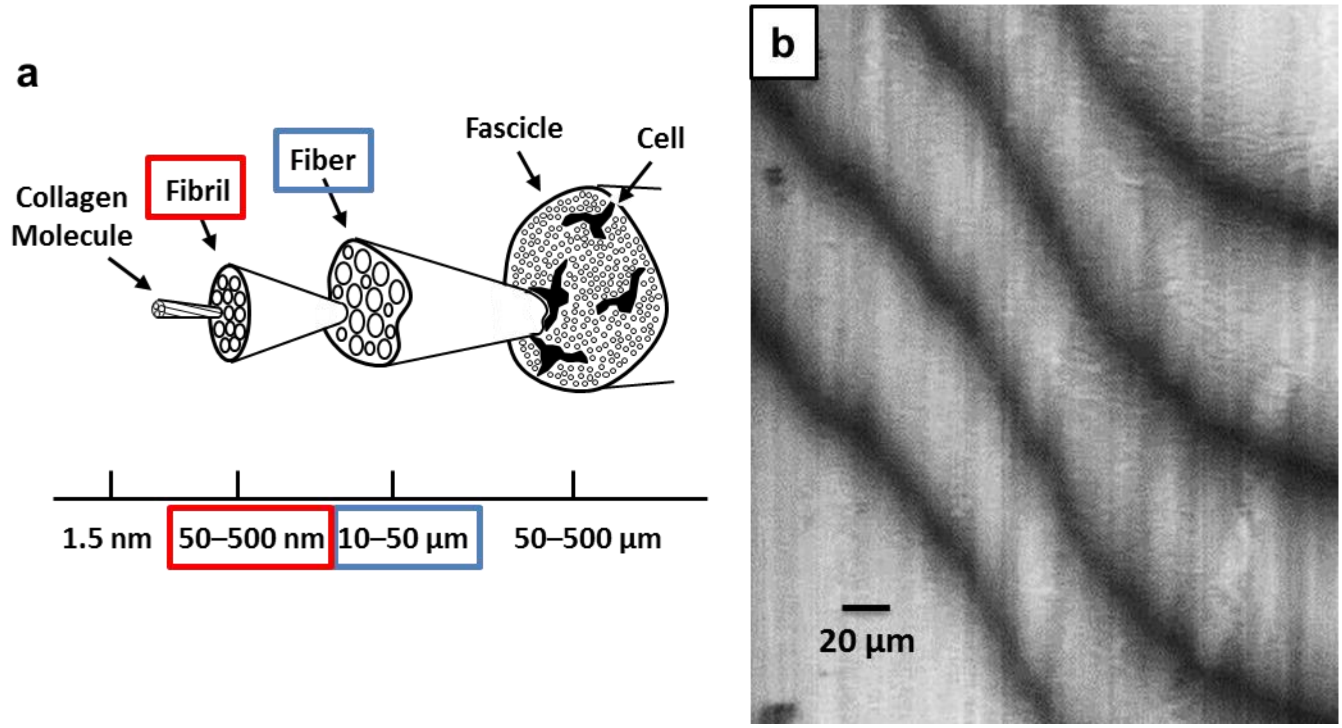

Supplementary Figure 6. Length scale of structural origins of shear stress. (a) Within tendon fascicles, the fibrils are smaller than the resolution of the optical microscope (820 nm). However, bundles of fibrils (i.e., fibers) are large enough to be visible in the microscopic images. (b) Prior to the formation of discontinuities at the cut/uncut tissue interface, the photobleached lines were continuous down to the limiting resolution of the microscope. This suggests that the shear strains (and hence the shear stress) occur at a length scale below the imaging resolution (i.e., between individual fibrils) and not simply at the fiber boundaries. Figure drawn by SES.

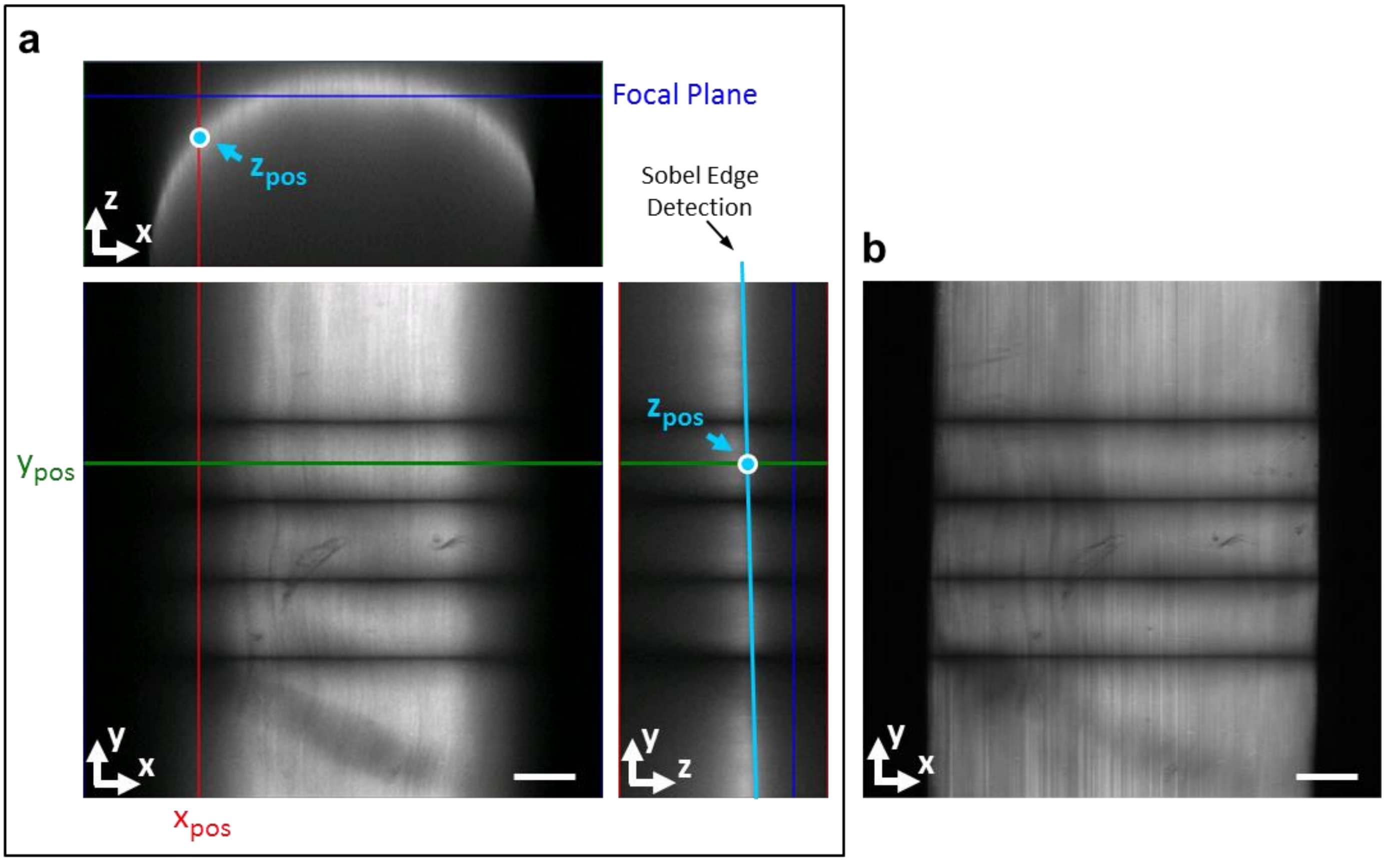

Supplementary Figure 7. Flattening of image stack to a single composite image spanning the full tissue width. (a) Due to the curved surface of tendon fascicles, no single focal plane contained an image spanning the full tissue width. Therefore, images stacks were obtained through the sample depth. At a every x-position within the image stack, a Sobel edge detection algorithm was applied to the y,z-plane. The resulting (cyan) line indicated the position of the sample surface (z_{pos}) as a function of the y-position in the image stack. (b) To generate the flattened image, the two focal planes bounding the sample surface (z_{pos}) at every x,y pixel location were determined. The intensity values of these images at the given x,y pixel location were averaged producing the single flattened image spanning the full tissue width. Scale bars, 100 µm.

Supplementary Methods

Testing of gelatin gel. *Sample preparation.* To fabricate the gel, a 20% (w/v) solution of porcine skin gelatin (Sigma-Aldrich) and deionized water at 55 °C was poured into a Teflon mold at room temperature. Upon solidification, the gel construct was removed from the mold and crosslinked with 0.05% glutaraldehyde in phosphate buffered saline (PBS, pH 7.4) for 22 h in order to prevent swelling and stabilize the mechanical properties[29]. The gel was then fluorescently stained with a 10 μg/ml solution of 5-(4,6-dichlorotriazinyl)aminofluorescein (Life Technologies) in 0.1 M sodium bicarbonate buffer (pH 9.0) for 20 min at room temperature with gentle shaking. The gel was washed in PBS and a 0.75 mm biopsy punch was used to produce a semi-circular notch approximately 4.5 mm from the gel midpoint. The gel was then loaded into the PBS bath of a custom uniaxial testing device mounted on an inverted confocal microscope[5] (LSM 5 LIVE; 10x C-Apochromat water immersion lens, Zeiss).

Mechanical Testing. Prior to testing, a 1 mN preload was applied to define the reference length (33.0 mm). A series of line scans were captured through the sample thickness to determine the cross-sectional dimensions (540 μm thick x 680 μm wide). Sets of four lines, with each line 2 μm wide and separated by 100 μm, were photobleached onto the gel surface with a laser diode (489 nm, 100 mW, 100 iterations of 100 μs) at 0, 0.4, 0.8, 1.2, 2.0, and 4.0 mm from the notch midline. At each location, images (0.82 x 0.82 μm/pixel) were taken of the photobleached lines to determine their reference positions. Grip-to-grip strains were then applied to the gel in 4% strain increments at 0.05%/s and held for 5 min before imaging the photobleached lines at each location. This was repeated to a total grip-to-grip strain of 20%. The applied load was continuously recorded with a 10 N load cell (Model 31, Honeywell).

Data analysis. A custom Matlab algorithm was used to find the pixel locations of the photobleached lines in each image at all locations. Briefly, at a given position along the x-direction, the y-position of each line was determined by the pixel with the minimum signal intensity. These values were smoothed by computing a moving average across each line with an averaging window of 35 pixels. At each x-position and between each pair of lines, axial strains (ε_{yy}) were calculated as the change in the distance between line pairs compared to

their positions at 0% applied strain. The Young's modulus of the gel (E = 585 kPa) was determined by performing a least-squares fit of the line $\sigma = E\varepsilon$, where σ is the applied stress and ε is the average axial strain at the 4 mm location measured for each grip-to-grip strain increment (R^2 = 0.993). Note that 4 mm was suitably far-field from the notch so that the axial strains at this location were uniform (Fig. 1c).

Finite element analysis. To assess the accuracy of the experimental strain measurements, we conducted a finite element simulation of the gel notch tension testing. The model was constructed within SolidWorks (Dassault Systemes) with the same geometry as the imaging area of the physical gel sample and composed of triangular two-dimensional shell elements. A fixed constraint was applied at a distance 5 mm from the notch and a 0.4 mm displacement was applied to the notch midline, simulating an applied tensile strain of 8%. The material was defined as isotropic, linear elastic, and incompressible with a Young's modulus of 585 kPa. The resulting axial strain field was compared graphically to the experimental measurements taken at a grip-to-grip strain of 8%.

Testing of tendon fascicles. *Sample preparation.* Eight fascicles were harvested from five tails of 7-month-old Sprague-Dawley rats sacrificed for a separate IACUC approved study. Each fascicle was cut to a length of 45 mm and fluorescently stained with a 10 µg/ml solution of 5-(4,6-dichlorotriazinyl)aminofluorescein (Life Technologies) in 0.1 M sodium bicarbonate buffer (pH 9.0) for 20 min at room temperature with gentle shaking. Previous testing of intact samples demonstrated that staining at this concentration has a minimal effect on tendon fascicle mechanics[15]. The samples were washed in PBS and placed into the same uniaxial device used to test the gel construct. Only fascicles with a clear elliptical shape were chosen for testing and care was taken to ensure that the samples were not twisted when clamped in the grips of the testing device. To measure the tissue cross-sectional area, the ends of each sample were rotated by 360° immediately before testing. Images of the sample profile using reflected light (633 nm) were obtained with the microscope at the points along the sample length where the major axis was in a horizontal or vertical orientation. These images were used to determine the major and minor diameters, and the cross-sectional area was calculated assuming the samples were elliptical

(0.219 ± 0.025 mm^2). The samples were then rotated back into a straight orientation and tested as described below.

Mechanical Testing. A 1 mN preload (~5 kPa) was applied to the tissue to define the reference length (31.9 ± 0.1 mm). The sample was preconditioned by applying five cycles of 2% grip-to-grip strain at 0.5%/s and then allowed to recovery at the reference length for 10 min. After the recovery period, the sample was partially transected with a scalpel blade 8.7 ± 0.3 mm from either the left or right grip producing a sharp notch extending 48 ± 13% across the sample width. The notch was placed near each grip for an equal number of samples to ensure that the measured strain gradients were due to the presence of the notch and not gripping artifacts. Sets of four lines, with each line 2 μm wide and separated by 125 μm, were photobleached onto the tissue surface with a laser diode (489 nm, 100 mW, 300 iterations of 100 μs) at 0, 4, 8, and 12 mm from the notch. In order to photobleach the entire tissue width, line scans were performed at each location through the tissue depth in 5 μm increments. Microscale image stacks (0.82 x 0.82 x 3.7 μm/pixel) were taken of the initial positions of the photobleached lines. This was necessary since the curved surface of the fascicles meant that no single focal plane contained an image spanning the full tissue width. Grip-to-grip strains were then applied in 1% strain increments at 0.05%/s and held for 5 min before imaging the photobleached lines at each location. This was repeated to a total grip-to-grip strain of 8%.

Image processing and data analysis. After testing, the microscale image stacks were flattened to a single image of the full tissue width using a custom Matlab algorithm. At every x-position within the image stack, a Sobel filter was used to determine the edge of the sample surface in the two-dimensional image constructed from the y,z-plane (Supplementary Fig. 7a). A least-squares linear fit was applied to the detected edge, which was used to determine the z-position of the sample surface as a function of the y-position. Using this information at every pixel location in the x,y-plane, the flattened image was generated by computing the weighted average of the intensity values at the corresponding pixel in the two images bounding the specified z-position (Supplementary Fig. 7b). Using this flattened image, the pixel locations of the photobleached lines and the axial strains (ε_{yy}) were calculated as described for the gel construct.

Calculation of axial stress gradient and interfibrillar shear stress. Based on the axial force equilibrium equation $\partial\sigma_{yy}/\partial y + \partial\sigma_{xy}/\partial x = 0$, an estimate for the interfibrillar shear stress (σ_{xy}) was calculated from the axial stress gradient ($\partial\sigma_{yy}/\partial y$) on the uncut side of the fascicle. The boundary of the uncut side was determined as the x-position of the initial discontinuity within the photobleached lines at each imaging location (Fig. 2a). These positions were also used to determine the propagation angle of the discontinuities along the sample length with respect to the fascicle axis (Supplementary Fig. 2). Assuming that the uncut side was a constant fraction of the fascicle width throughout testing, the position of the uncut boundary at earlier points during testing was determined at each imaging location using the width fraction computed from the initial appearance of discontinuity. To reduce the effect of noise on the shear stress calculation, the axial strains were averaged across the width of the uncut side for every applied grip-to-grip strain value and at every imaging location. Assuming that only the axial strains of the fibrils contribute to the axial stress within the fascicle and neglecting any contribution from transverse or shear strains of the matrix[16,25], the average axial strain on the uncut side was converted to stress using an empirical relationship between the microscale fibril strains and applied stress from previous testing of intact rat tail tendon fascicles using the same testing protocols[15] (Supplementary Fig. 3). To evaluate the stress gradient within each sample, the stress values were normalized to the location 4 mm away from the notch. Normalized values statistically different from one were determined by one sample Student's t-tests with significance set at $p \leq 0.05$.

The interfibrillar shear stress was calculated as the average shear stress acting at the interface between the cut and uncut sides of the tissue. Shear stress values (σ_{xy}) were computed using the axial force balance $\sigma_{xy}A_x = \Delta\sigma_{yy}A_y$, where $\Delta\sigma_{yy}$ is the decrease in axial stress between the 4 mm and 8 mm locations or the 8 mm and 12 mm locations at each grip-to-grip strain, A_x is the interfacial area between the cut and uncut sides, and A_y is the average uncut cross-sectional area (Supplementary Fig. 5a). This force balance can be derived directly from the axial force equilibrium equation ($\partial\sigma_{yy}/\partial y + \partial\sigma_{xy}/\partial x = 0$) as follows. Given the assumption stated above, the axial stress is a function of only the fibril strains ($\sigma_{yy} = f(\varepsilon_{yy}(y))$), where $f(\varepsilon_{yy}(y)$ is given by Supplementary Fig. 3. Substituting this into the equilibrium equation produces the partial differential equation

$$\partial\sigma_{xy}(x,y)/\partial x = -\,df\,(\varepsilon_{yy}(y))/dy\ . \qquad (1)$$

We then assumed that, at a given x-position, the shear stress was constant between each pair of imaging locations (e.g., between the 4 mm and 8 mm locations or between the 8 mm and 12 mm locations) and eliminated the partial derivative of σ_{xy} by splitting equation (1) into the following pair of equations

$$d\sigma_{xy}(x,y_i)/dx = -\,df\,(\varepsilon_{yy}(y_i))/dy \quad i = 1, 2 \qquad (2)$$

where y_i represents the midpoint between the 4 mm and 8 mm locations or between the 8 mm and 12 mm locations. Defining Δx as the uncut sample width (in the undeformed reference configuration), Δy as the distance between the imaging locations (in the undeformed reference configuration), and given the boundary condition $\sigma_{xy}(\Delta x,y_i) = 0$, we obtained the finite difference approximation of

$$[\ \sigma_{xy}(\Delta x,y_i)\ -\ \text{-}\sigma_{xy}(0,y_i)\ ]\ /\ \Delta x = \sigma_{xy}(0,y_i)\ /\ \Delta x = -\,[\ f\,(\varepsilon_{yy}(y_i^{+}))\ -\ f\,(\varepsilon_{yy}(y_i^{-}))\ ]\ /\ \Delta y \qquad (3)$$

where y_i^{+} is the imaging loacation further from the notch and y_i^{-} is the imaging location closer to the notch for each pair of imaging locations. (Note: A shear stress $\sigma_{xy}(0,y_i)$ in the positive y-direction is actually a negative value since it is acting on the sample face in the negative x-direction). Both sides of this equation were multiplied by Δx and Δy and Δz, which is the thickness of the sample at $x = 0$, which produces

$$\sigma_{xy}(0,y_i)\ \Delta y\ \Delta z = -\,[\ f\,(\varepsilon_{yy}(y_i^{+}))\ -\ f\,(\varepsilon_{yy}(y_i^{-}))\ ]\ \Delta x\ \Delta z\ . \qquad (4)$$

Noting that $\Delta y\Delta z$ is the interfacial area between the cut and uncut sample sides between the imaging locations (Ax) and replacing $\Delta x\Delta z$ with the intact cross-sectional area (Ay) results in

$$\sigma_{xy}(0,y_i)\ Ax = \Delta\sigma_{yy}(y_i)\ Ay \qquad (5)$$

where $\Delta\sigma_{yy}(y_i) = -\,[\ f\,(\varepsilon_{yy}(y_i^{+}))\ -\ f\,(\varepsilon_{yy}(y_i^{-}))\ ]$, which represents the decrease in the axial stress along the sample length between each pair of imaging locations. Values for $\sigma_{xy}(0,y_i)$ were calculated at each grip-to-grip strain up to the initiation of failure at the cut/uncut tissue interface (i.e., 4% grip-to-grip strain) and were averaged to produce a single value for the interfibrillar shear stress acting within each sample.

Tracking of fibril trajectories. *Sample preparation.* One fascicle was harvested from the tail of a 7-month-old Sprague-Dawley rat and cut into segments approximately 10 mm long. The segments were processed according

to a protocol specifically developed for tracking collagen fibrils in tendon using serial block-face scanning electron microscopy (SBF-SEM)[30]. Briefly, the fascicle segments were fixed in 2.5% (v/v) glutaraldehyde in 100 mM cacodylate buffer (pH 7.4) at 4 °C overnight. The segments were washed and then incubated in 2% (v/v) osmium tetroxide and 1.5% (w/v) potassium ferrocyanide in the cacodylate buffer for 1 h at room temperature (RT). The segments were washed with distilled water and stained with 1% (w/v) tannic acid in the cacodylate buffer two times for 2 h at 4 °C. The segments were then stained a second time with 2% (v/v) osmium tetroxide in distilled water for 40 min at RT. Following a wash in distilled water, the segments were incubated in 1% (w/v) uranyl acetate (aqueous) for 13.5 h at 4 °C and again washed. The segments were then dehydrated in serial ethanol solutions of 25%, 50%, 75%, and 95% (v/v) with distilled water for 10 min each, followed by four 10 min incubations in 100% ethanol. The segments were transferred to propylene oxide for 10 min at RT and infiltrated in a graded series of EMbed 812 Hard resin (Electron Microscopy Sciences) in propylene oxide (v/v) at RT: 30% for 4 h, 50% overnight, 70% and 90% for 1 h each, and 100% three times for 1 h. Finally, the segments were cured in 100% EMbed 812 Hard resin for 72 h at 60 °C.

Scanning electron microscopic imaging. The resin block was positioned with the fascicle axis oriented vertically and then faced to expose the tissue cross-section. At a single location, 88 serial cross-sectional images (2000 x 2000 pixels, 5 nm/pixel) were taken every 100 nm along the fascicle length with a Merlin VP Compact scanning electron microscope (Zeiss) fitted with a 3View2XP *in situ* ultramicrotome (Gatan). Microscope conditions were 10 kV, 60 Pa (in VP mode), 10 μs dwell time, and a 60 μm aperture in high current mode for probe selection.

Image processing. Processing of the raw image stack was performed using Fiji[31] to improve image quality and allow for automated tracking of fibril trajectories. A rigid alignment was performed to correct for a small drift in the field-of-view with each image slice. The images were smoothed with a 3 pixel Gaussian filter and a rolling-ball background subtraction (diameter 300 pixels) was used to reduce general intensity fluctuations between images. The images were then converted into a binary format using the default thresholding algorithm. A watershed procedure was applied to separate objects that had fused during the

threshold operation. Finally, the stack was cropped to a consistent field-of-view across all image slices (final dimensions: 1662 x 1632 x 88 pixels).

Fibril Tracking. A custom Matlab algorithm was used to identify the fibrils in each image and track their trajectories through the image stack. First, in each image, every object larger than 20 pixels and not touching the image border was identified. All objects in the first image were defined as fibrils. In each subsequent image, any object whose centroid was within the radius of a fibril from the previous image was associated with that fibril. If more than one object fit this criterion for a fibril, only the closest object was chosen. To prevent early truncation of fibrils due to objects missing from a single image because of variations in thresholding or object separation, the algorithm also associated objects with centroids within the radius of a fibril from the second previous image. However, this only occurred if no object had been associated with the same fibril in the intervening image and if the object had a similar area to the fibril. The algorithm prevented any duplicate assignments and any unassociated objects were defined as new fibrils. Objects that were not fibrils (e.g., cellular material) were removed and minor corrections to fibril associations were manually made via visual inspection of labeled images produced by the algorithm. Finally, only fibrils that spanned a minimum of 10 image slices (1 μm) were included in subsequent analyses, resulting in a total of 1,213 individually tracked fibrils.

The diameter of each fibril was determined by calculating the diameter of a circle with the same area in each image and averaging over all images within the stack. The angle of the fibrils with respect to the fascicle axis was calculated by the inverse tangent of the distance between the first and last centroid locations divided by the length along the fascicle axis spanned by the fibril. Note that the direction of the fibril trajectories was not considered in this calculation; therefore, all angle values were positive in magnitude. A linear correlation was used to assess the relationship between fibril angle and diameter.
